# Immunopharmacological potential of *Arctium lappa* L. in immune-mediated skin diseases: A critical review of experimental and clinical evidence

**DOI:** 10.3389/fphar.2025.1660352

**Published:** 2025-10-03

**Authors:** Mengyao Yang, Ge Peng, Alafate Abudouwanli, Shan Wang, Quan Sun, Wanchen Zhao, Yi Tan, Xuefei Du, Li Zhang, Hideoki Ogawa, Ko Okumura, Xinghua Gao, François Niyonsaba

**Affiliations:** ^1^ Department of Dermatology, The First Hospital of China Medical University, Shenyang, China; ^2^ Atopy (Allergy) Research Center, Juntendo University Graduate School of Medicine, Tokyo, Japan; ^3^ Department of Dermatology, National Center for Children’s Health, Beijing Children’s Hospital, Capital Medical University, Beijing, China; ^4^ Faculty of International Liberal Arts, Juntendo University, Tokyo, Japan

**Keywords:** *Arctium lappa* L., arctigenin, arctiin, immune-mediated inflammatory disease, immune-mediated skin diseases

## Abstract

**Background:**

*Arctium lappa* L. (*A. lappa*) has been used in traditional medicine worldwide and is increasingly being investigated for its immunomodulatory and anti-inflammatory effects. However, its therapeutic relevance for immune-mediated skin diseases (IMSDs) remains incompletely defined.

**Objective:**

This review critically evaluates experimental and clinical evidence on *A. lappa* and its major lignans, arctiin and arctigenin, in IMSDs, including those associated with atopic dermatitis (AD), psoriasis, systemic lupus erythematosus (SLE), alopecia, systemic sclerosis (SSc), and vasculitis.

**Methods:**

We systematically searched PubMed, Web of Science, and Scopus up to July 2025 using defined keywords. Eligible studies included *in vitro*, *in vivo*, and clinical investigations assessing the immunological and dermatological outcomes of *A. lappa* extracts or purified metabolites.

**Results:**

Preclinical studies have demonstrated that *A. lappa* extracts and their lignans modulate key inflammatory pathways, including the NF-κB, JAK/STAT, and NLRP3 inflammasome signaling pathways. Evidence indicates protective effects on keratinocyte hyperproliferation, mast cell activation, dermal fibroblast fibrosis, and vascular endothelial inflammation. However, most data are derived from *in vitro* or murine models using heterogeneous preparations, with limited clinical validation. Reported doses range from 10–100 μM in cell assays to 15–100 mg/kg in animal studies, but pharmacokinetic and safety data remain insufficient.

**Conclusion:**

*A. lappa* shows promising immunopharmacological potential for IMSDs, but the evidence remains preliminary. The current literature is limited by variability in extract preparation, a lack of standardized dosing, and the absence of robust randomized clinical trials. Future research should prioritize standardized phytochemical characterization, translational animal models, pharmacokinetic studies, and controlled clinical investigations to establish efficacy and safety.

## 1 Introduction

In recent years, the immunomodulatory properties of medicinal plants have garnered extensive research interest because of their potential in treating immune-mediated diseases. To date, approximately 150 medicinal plants with immunomodulatory effects have been identified as promising sources for the development of new therapies. Notably, approximately 40% of the plants studied for their immunomodulatory effects belong to the *Asteraceae* family, including *Arctium lappa* L. (*A. lappa*), and at least 18 species within this family have documented immunomodulatory activities (Zebeaman et al., 2023). Given that *Asteraceae* is among the largest plant families globally, its high representation in immunopharmacological studies underscores its considerable therapeutic potential (Michel et al., 2020). Similar immunomodulatory activity is also observed in marine-derived peptides from Atlantic cod, underscoring the broad relevance of nature-based immunoregulators (Yuan et al., 2023).

Immune-mediated skin diseases (IMSDs), including conditions such as atopic dermatitis (AD), psoriasis, systemic lupus erythematosus (SLE), alopecia areata, systemic sclerosis (SSc), and vasculitis, represent a clinically diverse group of dermatological disorders characterized by chronic and recurrent inflammation caused by dysregulated immune responses (Diotallevi et al., 2022). Although significant therapeutic advancements—such as the use of corticosteroids, immunosuppressants, and biologic agents—have substantially improved disease management, many patients still experience limited treatment efficacy, adverse effects, and frequent relapses (Alaibac, 2023). Consequently, the demand for complementary therapeutic approaches, such as medicinal plant extracts that can enhance treatment outcomes while minimizing adverse effects, is increasing.

Among these medicinal plants, *A. lappa* (commonly known as burdock) has been widely studied. It has also been recorded in historical literature under several synonyms, including *Lappa major* Gaertn., *Lappa officinalis* All., and *Arctium majus* Bernh., all of which are validated by the Plants of the World Online (POWO) (Powo, 2025) (Supplementary Table S1). This perennial plant has a long-standing history of medicinal use in traditional Chinese medicine (Jin et al., 2023; Yosri et al., 2023), European folk medicine, and North American herbal practices (Sung et al., 2016; Ambroselli et al., 2024). The fruits and roots of *A. lappa*, in particular, are highly valued for their therapeutic properties and are widely used across Eurasia (Mei et al., 2024), whereas in North America, *A. lappa* is used both as a botanical drug and a functional food ingredient. A broad spectrum of pharmacological effects have been attributed to *A. lappa*, including anti-inflammatory (Ji et al., 2022; Mai et al., 2025), antioxidant (Hassanein et al., 2024; Liu et al., 2014), antiangiogenic (Taleb et al., 2020), antitumor (Lou et al., 2017), antiaging (Chen et al., 2024), neuroprotective (Wei et al., 2021; Yuan et al., 2022), memory-enhancing (Lee et al., 2011), gut microbiota-regulating (Wang et al., 2024), immunomodulatory (Li Y. et al., 2022; Zeng et al., 2024), and anti-constipation effects (Li et al., 2019).

Numerous studies have characterized the chemical composition and pharmacological effects of extracts of *A. lappa*, which is rich in diverse bioactive metabolites, such as lignans, polysaccharides (Li L. et al., 2021), phenolic acids (Ambroselli et al., 2024; Szparaga et al., 2021), volatile oils, phytosterols, flavonoids, and fatty acids, all of which contribute to its wide-ranging health benefits (Li L. et al., 2022). Among these metabolites, lignans such as arctiin and arctigenin, the major bioactive metabolites of Fructus Arctii (*A. lappa* fruit) (Jin et al., 2023), have been recognized for their broad pharmacological activities across multiple plant species (Su et al., 2023). Extensive *in vitro* and *in vivo* studies have shown that arctiin and arctigenin have anti-obesity (Zeng et al., 2023), antioxidant (Jia et al., 2024), antitumor (Hausott et al., 2003; Romualdo et al., 2020), anti-inflammatory (Alhusaini et al., 2019), antidiabetic, anti-allergic, gastroprotective, and neuroprotective effects (Li et al., 2016).

The diverse therapeutic properties of *A. lappa* underscore its importance in both traditional and modern medicine. The botanical characteristics, traditional uses (Li Y. et al., 2022), chemical constituents, pharmacological effects (Li Z. et al., 2024), ethnopharmacology, quality control methods, phytochemistry, derivatives, and toxicity of *A. lappa* have been systematically addressed (Jin et al., 2023).

Given the increasing incidence of IMSDs and the limitations of currently used therapies, a comprehensive review focusing on the anti-inflammatory and immunomodulatory properties of *A. lappa* is warranted. In this study, the potential of *A. lappa* as a complementary therapeutic agent is discussed, offering alternative strategies for patients who experience inadequate responses or adverse effects with conventional treatments.

## 2 Methods—literature search strategy

A systematic literature search was performed in PubMed, Web of Science, Scopus, ScienceDirect, and Google Scholar up to July 2025. The following search terms were used in different Boolean combinations: “Arctium lappa” OR “burdock” OR “Fructus Arctii” OR “Arctigenin” OR “Arctiin” OR “phytochemical” AND “immune” OR “inflammation” OR “immunomodulation” OR “skin” OR “psoriasis” OR “atopic dermatitis” OR “systemic lupus erythematosus” OR “alopecia” OR “systemic sclerosis” OR “vasculitis.” In parallel, ethnopharmacological records were retrieved from authoritative traditional medicine sources, such as *Shennong Bencao Jing*, *Bencao Gangmu*, *Mingyi Bielu*, *the Pharmacopoeia of the People’s Republic of China*, and Japanese references, including *Wakan–Yaku Jiten* and *Nihon Hanpō Igaku*. Eligible studies included original *in vitro*, *in vivo*, or clinical research reporting immunomodulatory, anti-inflammatory, or dermatological effects of *A. lappa* or its major lignans. The exclusion criteria were nonoriginal works, studies without immunological relevance, or those unrelated to immune-mediated diseases. Reference lists of relevant reviews were also screened to identify additional eligible studies. All included information was manually curated and categorized by theme to support a structured narrative analysis across each section of the review. All cited pharmacological studies involving *Arctium lappa* were taxonomically validated using the POWO database and the Medicinal Plant Names Services (MPNS) portal. In addition, all other cited plant species [e.g., *Forsythia suspensa* (Thunb.) Vahl, *Lonicera japonica* Thunb., *Isatis indigotica* Fortune, *Taraxacum officinale* F.H. Wigg., and *Melissa officinalis* L.] were likewise cross-checked and validated against POWO/MPNS to ensure full taxonomic accuracy and consistency.

## 3 Arctiin and arctigenin: key lignans in *A. lappa*

The total lignan content of *A. lappa* has been well characterized, with arctiin identified as the most abundant lignan (Zhang J. Y. et al., 2022). This bioactive metabolite exhibits a broad spectrum of pharmacological activities, notably anti-inflammatory, antioxidant, and anticancer (Lee et al., 2019; Li et al., 2017a). Arctiin effectively alleviates the cellular dysfunction and oxidative stress induced by reactive oxygen species (ROS), highlighting its potential to protect against cellular damage (Bae et al., 2014; Su et al., 2015). One of the key mechanisms of action of arctiin is its ability to modulate inflammatory signaling pathways. In models of chronic stress-induced neuronal injury, the expression of the purinergic receptor P2X_7_ (P2X_7_R) is significantly upregulated, triggering the activation of downstream inflammasome components. Treatment with arctiin markedly downregulates P2X_7_R expression, which, in turn, suppresses the activation of the NOD-like receptor family pyrin domain-containing 3 (NLRP3) inflammasome. This reduction in NLRP3 expression is accompanied by decreased cleavage of caspase-1 and maturation of interleukin (IL)-1β, suggesting the effective inhibition of inflammasome-mediated inflammation. In parallel, arctiin suppresses the activation of toll-like receptor (TLR) 4 and nuclear factor kappa-light-chain-enhancer of activated B cells (NF-κB), as evidenced by the decreased nuclear translocation of NF-κB and the subsequent downregulation of pro-inflammatory cytokines (Zhou et al., 2018). In addition to its anti-inflammatory properties, arctiin also attenuates neuroinflammation (Xu et al., 2020), further underscoring its therapeutic potential in IMSDs.

Arctigenin, the aglycone form of arctiin, a bioactive dibenzylbutyrolactone lignan found in *A. lappa*, is recognized as an effective inhibitor of mitogen-activated protein kinases (MAPKs), including extracellular signal-regulated kinases 1 and 2 (ERK1/2), p38 kinase, and Jun N-terminal kinase (JNK) (Cho et al., 2004). In addition, arctigenin targets key signaling molecules, such as phosphoinositide 3-kinase (PI3K), protein kinase B (AKT), and mechanistic target of rapamycin (mTOR). By decreasing the levels of their phosphorylated forms without altering total protein levels, arctigenin effectively suppresses cell proliferation, invasion, and epithelial–mesenchymal transition (Zhou et al., 2018). Conversely, arctigenin activates the adenosine monophosphate-activated protein kinase (AMPK) pathway, which is crucial for maintaining cellular energy homeostasis and contributes to its anti-inflammatory and antitumor effects (Xu et al., 2020). These dual actions, namely, simultaneous inhibition of the PI3K/AKT/mTOR axis and activation of AMPK signaling, position arctigenin as a promising candidate for the treatment of inflammation, metabolism, and cancer progression (Chen X. F. et al., 2024).

Pharmacokinetic studies have indicated that the oral delivery of arctiin and arctigenin presents significant challenges because of their limited absorption and extensive metabolism. Arctiin, a lignan glycoside, undergoes hydrolysis in the gut to release arctigenin, which is more lipophilic and better absorbed. However, arctigenin itself is rapidly metabolized via glucuronidation and sulfation, resulting in low systemic exposure (Gao et al., 2018). Animal studies have shown its distribution to the liver, kidney, and inflamed tissues, but data from humans are scarce (Li et al., 2017b). Both metabolites are eliminated primarily as conjugates through urine and bile (Wang et al., 2013; He et al., 2019). The structural differences between glycosides (arctiin) and aglycones (arctigenin) strongly influence their absorption, distribution, metabolism, and excretion.

A comparative analysis of arctiin and arctigenin concentrations across various tissues of *A. lappa* is provided in Table 1, highlighting the distributions and potential yields of these therapeutic lignans.

**TABLE 1:** Structural information, derivatives, and phytochemical distribution of arctiin and arctigenin in *A. lappa*

Metabolite	Content w/w	Part of *A. lappa*	Molecular structure	Derivative
Arctiin	2%–10%	Seeds	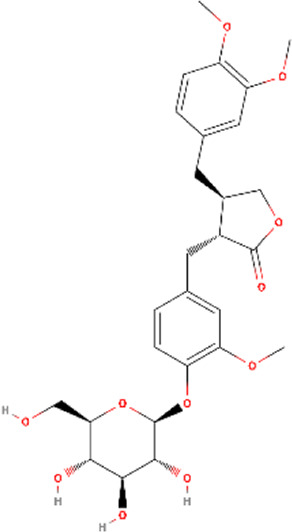	Arctigenin-4′-O-glucoside Arctiin-4′-O-gentiobioside Arctiin acetate Arctiin methyl ether Arctiin sulfate Arctiin phosphate
	0.04%	Roots		
	0.06%	Stems		
	0.05%	Flower		
	0.00338%	Leaves		
Arctigenin	0.5%–2%	Seeds	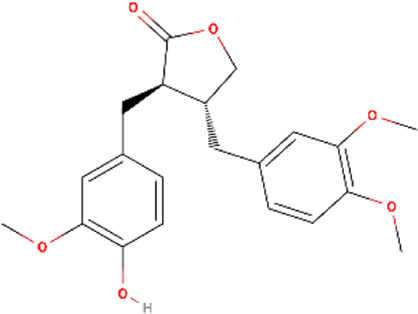	5-Hydroxyarctigenin Arctigenin quinone Arctigenin-4′-O-glucoside Arctigenin monoacetate Arctigenin methyl ether Arctigenin trans-(+)-arctigenin 4′-O-beta-gentiobioside
	0%–0.00127%	Roots		
	0.05%	Flower		
	0.00197%	Leaves		

## 4 Arctiin and arctigenin in immune regulation

**FIGURE 1:**
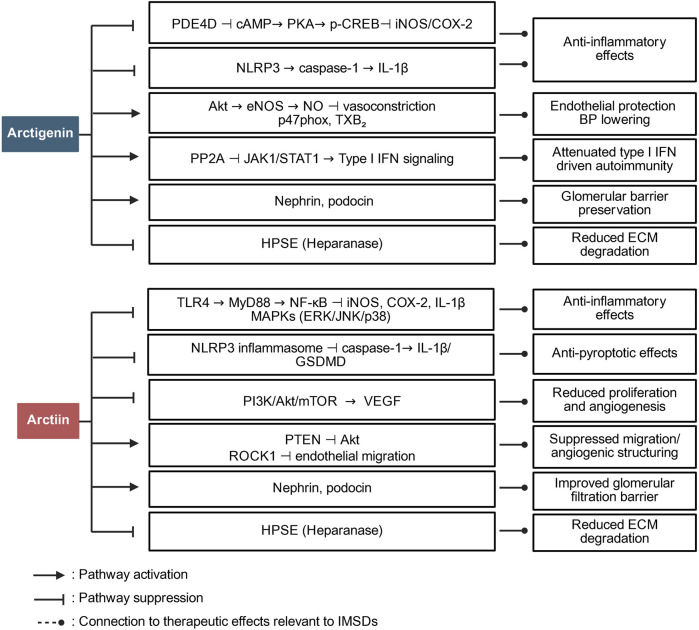
Pathway overview of the role of arctiin and arctigenin in immune regulation. The figure summarizes how arctigenin and arctiin modulate major inflammatory and immune-relevant signaling networks in IMSDs.

Preclinical studies have indicated that arctiin and arctigenin exert disease-related immunomodulatory effects, including inhibition of NF-κB activation, suppression of pro-inflammatory cytokine release, and modulation of oxidative stress (Liu et al., 2021); thus, these compounds are promising candidates for managing immune-mediated inflammatory conditions. Figure 1 summarizes the major targets and pathways modulated by arctiin and arctigenin.

Arctiin exerts its immunomodulatory effects primarily by suppressing key inflammatory mediators, including TLR4, myeloid differentiation primary response 88 (MyD88), and NF-κB, which play central roles in the regulation of inflammatory responses. This inhibition leads to decreased production of pro-inflammatory cytokines such as IL-1β and interferon (IFN)-γ; by attenuating the activation of NLRP3, caspase-1, and gasdermin D (GSDMD), arctiin inhibits pyroptosis—a form of programmed inflammatory cell death—thereby mitigating the immune hyperactivation observed in diseases such as AD (Li J. et al., 2024). Molecular docking studies further confirmed that arctiin can directly bind to TLR4, inhibiting its activation and downstream signaling.

Arctigenin, the bioactive derivative of arctiin, has additional immunomodulatory properties, notably through the modulation of the gut microbiota—a critical metabolite in systemic immune regulation. Arctigenin promotes the growth of short-chain fatty acid-producing bacteria, which subsequently activate G protein-coupled receptors 41 and 43 (GPR41/43) and inhibit histone deacetylase 3 (HDAC3), thereby contributing to the maintenance of the balance between T helper 17 (Th17) cells and regulatory T (Treg) cells—an essential mechanism for controlling inflammation and autoimmunity (Wang et al., 2024). Moreover, arctigenin suppresses the production of lipopolysaccharide (LPS), a potent inflammatory stimulus, by inhibiting gut-derived TLR4/NF-κB activation, thereby attenuating systemic inflammation (Lu et al., 2020). In addition to modulating cytokine signaling and gut–immune interactions, arctigenin influences macrophage polarization. It promotes the anti-inflammatory M2 phenotype while inhibiting the pro-inflammatory M1 phenotype (Wang et al., 2024), facilitating the resolution of chronic inflammation, a crucial process in diseases such as rheumatoid arthritis and inflammatory bowel disease.

Moreover, both arctiin and arctigenin exhibit antioxidant properties by scavenging ROS, reducing oxidative stress, and enhancing cellular resistance to inflammatory damage (Chen et al., 2024; Liu et al., 2021; Li G. et al., 2022; Chen et al., 2020). These antioxidant mechanisms further support the protective roles of these metabolites in inflammatory and immune dysregulation contexts. Preclinical and clinical studies have highlighted the therapeutic potential of these metabolites for a range of immune-mediated diseases. For example, arctiin exerts neuroprotective effects on neuroinflammatory conditions, such as depression, by regulating the P2X_7_R/NLRP3 inflammasome pathway (Jin et al., 2024). Similarly, arctigenin reduces systemic inflammation in individuals with metabolic disorders and restores intestinal and hepatic immune homeostasis (Wang et al., 2024).

Collectively, the available evidence suggests that arctiin and arctigenin act on multiple immunoinflammatory pathways. Their roles extend beyond general antioxidant activity to include targeted regulation of cytokine signaling, inflammasome activation, and fibroblast function.

## 5 Effects of *A. lappa* extracts on IMSDs

Immune-mediated inflammatory diseases (IMIDs) encompass a broad group of chronic disorders characterized by dysregulated immune responses and persistent inflammation. IMIDs can affect multiple organ systems, including the skin, joints, gastrointestinal tract, and central nervous system. IMSDs include clinically important conditions such as AD, psoriasis, SLE, alopecia areata, SSc, and vasculitis. The relationships between IMIDs and IMSDs, along with examples of common diseases in each category, are shown in Table 2.

**TABLE 2:** Overview of system-based IMID categories highlighting IMSD.

IMSD	Cardiovascular IMID	Pulmonary IMID	Gastrointestinal IMID	Endocrinological IMID
• AD • Psoriasis • SLE • AA • SSc • CV	• GCA • TA • Antiphospholipid syndrome • Myocarditis	• Sarcoidosis • ANCA-associated vasculitis • Goodpasture’s disease	• IBD • AIH • PBC • PSC	• AITD • Addison’s disease • APS • IgG4-related hypophysitis • AIP

Rheumatological IMID	Hematological IMID	Renal IMID	Neurological IMID	Ocular IMID
• RA • SS • AS • PsA • PM	• Evans syndrome (AIHA and ITP) • Autoimmune neutropenia • Autoimmune aplastic anemia	• Lupus nephritis • Anti-GBM disease • IgA vasculitis	• MS • NMOSD • AE • MG	• Uveitis • Scleritis • IgG4-related ophthalmic disease • MOG-ON

AE, autoimmune encephalitis; AIH, autoimmune hepatitis; AIHA, autoimmune hemolytic anemia; AIP, autoimmune pancreatitis; AITD, autoimmune thyroid disease; ANCA, anti-neutrophil cytoplasmic antibody; anti-GBM disease, anti-glomerular basement membrane disease; APS, autoimmune polyglandular syndrome; AS, ankylosing spondylitis; CV, cutaneous vasculitis; giant cell arteritis; IBD, inflammatory bowel disease; IgG4, immunoglobulin G4; ITP, immune thrombocytopenia; MG, myasthenia gravis; MOG-ON, myelin oligodendrocyte glycoprotein-associated optic neuritis; MS, multiple sclerosis; NMOSD, neuromyelitis optica spectrum disorder; PBC, primary biliary cholangitis; PM, polymyositis; PsA, psoriatic arthritis; PSC, primary sclerosing cholangitis; RA, rheumatoid arthritis; SS, Sjögren’s syndrome; TA, Takayasu arteritis.

IMSDs share overlapping pathogenic mechanisms, including abnormal cytokine production, chronic immune activation, and barrier dysfunction, although disease-specific variations exist. Genetic predisposition, environmental factors, and immune dysregulation together contribute to the tissue-damaging inflammation characteristic of these disorders. Conventional therapies, including corticosteroids, immunosuppressants, and biologics, have improved disease control but are often limited by insufficient efficacy, adverse effects, and variable patient responses (Monteleone et al., 2023).

**FIGURE 2:**
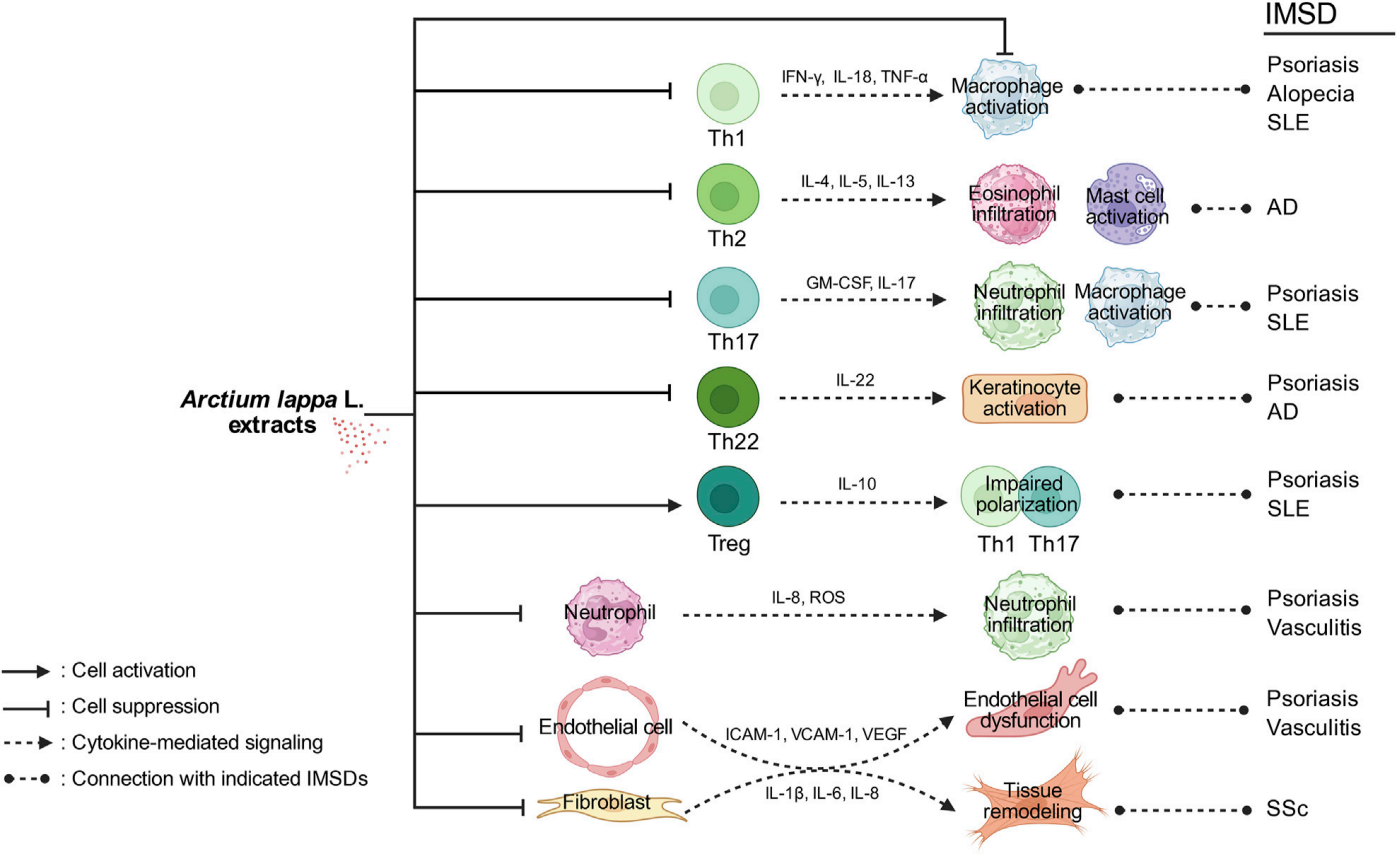
Schematic illustration of the effects of *Arctium lappa* L. extracts on IMSDs. *A. lappa* extracts modulate multiple immune and stromal cell responses involved in the pathogenesis of immune-mediated skin diseases. By suppressing Th1-derived cytokines such as IFN-γ, IL-18, and TNF-α, *A. lappa* extracts may reduce macrophage activation, which is implicated in psoriasis, alopecia, and systemic lupus erythematosus (SLE). The inhibition of IL-4, IL-5, and IL-13 production by Th2 cells may attenuate eosinophil infiltration and mast cell activation, which are the key features of atopic dermatitis (AD). The downregulation of IL-17 and GM-CSF expressions in Th17 cells can lead to reduced neutrophil infiltration and macrophage activation in psoriasis and SLE. Suppressing IL-22 production by Th22 cells may decrease keratinocyte activation, contributing to the control of inflammation in psoriasis and AD. Enhancing IL-10 production by Treg cells may impair the polarization and function of Th1 and Th17 cells, thereby limiting the inflammatory responses associated with psoriasis and SLE. By reducing the expressions of IL-8, reactive oxygen species (ROS), and adhesion molecules, *A. lappa* extracts may decrease neutrophil infiltration, which plays a role in cutaneous vasculitis. The inhibition of ICAM-1, VCAM-1, and VEGF expressions in endothelial cells may help alleviate endothelial dysfunction and vascular inflammation in vasculitis. Finally, the suppression of IL-1β, IL-6, and IL-8 in fibroblasts may contribute to reduced tissue remodeling, a pathological hallmark of systemic sclerosis (SSc). Together, these immunomodulatory effects illustrate the potential of *A. lappa* extracts in the treatment of IMSDs such as psoriasis, AD, SLE, vasculitis, and SSc.

Given the increasing incidence and substantial effects of IMSDs on patient quality of life, novel, safer, and more effective therapeutic strategies are urgently needed (Hyam et al., 2013; Wang et al., 2023). Natural metabolites such as arctiin and arctigenin, which exhibit anti-inflammatory, antioxidant, and immunomodulatory activities, have emerged as promising candidates (Chen X. F. et al., 2024). Preclinical studies have demonstrated the ability of arctiin and arctigenin to regulate cytokine production, inhibit inflammatory signaling pathways, promote tissue repair, and restore immune homeostasis, suggesting their therapeutic potential for IMSDs (Jin et al., 2024). An overview of studies on *A. lappa* extracts for the treatment of IMSDs is presented in Figure 2, while Table 3 provides a schematic illustration of the proposed mechanisms of action of these metabolites.

**TABLE 3:** Overview of studies using *A. lappa* extracts and their metabolites in IMSD.

IMSD	Metabolite	*In vitro*	*In vivo*	Route	Mechanism	Limitation	References
Psoriasis	Arctigenin	LPS-activated human PBMCs; RAW264.7 cells	Imiquimod-induced murine model	Topical 5% cream, once daily	↑cAMP/PKA and p-CREB ↓PDE4D, iNOS, and COX-2	Single dose only; no positive control	Li H. et al. (2021b)
AD	Arctiin	TNF-α/IFN-γ-induced HaCaT cells	DNCB-induced BALB/c mice	Oral 25 mg/kg, once daily	↓TLR4/MyD88/NF-κB; ↓NLRP3/Caspase-1/GSDMD	Single dose only; no dose or PK data; barrier and durability not evaluated	Li H. et al. (2021b)
AD	Matairesinol	MC/9 mouse mast cells	Topical DfE + 4% SDS-treated NC/Nga mice	Topical 50 μg/d (on ears) × 11 days	↓IL-4 and IgE levels ↑IFN-γ	No dose or PK data; lacked barrier assessment	Sung et al. (2016)
SLE	Arctigenin	IFN-α-treated B cells	MRL/lpr mice Imiquimod-treated lupus mice	15 mg/kg, three times/week × 12–18 weeks	↑PP2A ↓JAK1/STAT1; ↓IFN-I	No minimal effective concentration; single dose only; no PK data; renal functional recovery not assessed	Zhang X. et al. (2022)
Alopecia	Arctiin	H_2_O_2_-induced HHDPCs	N/A	N/A	↑Wnt/MAPK and miR-602/125a-5p/21-3p ↓ROS/miR-1290	*In vitro* only; no dose-response or PK data	Bae et al. (2014)
Vascular injury	Arctiin	HG-induced HRCECs	STZ-induced diabetic retinopathy rat model	Oral 1.38 and 0.69 g/kg/day × 12 weeks	↑ROCK1 and PTEN ↓PI3K/AKT/VEGF	No PK; no comparison to standard anti-VEGF	Zhou et al. (2020)
Vascular sclerosis	ALE	HUVECs	Atherosclerosis mouse	Oral 100 mg/kg/day × 4 days	↓IL-1β, IL-6, TNF-α, and MCP-1; ↓VCAM-1 and NF-κB signaling	*In vitro* active range reported without minimal effective concentration; no PK data	Lee et al. (2020)
Vascular sclerosis	ALR-S	HAECs	FeCl_3_-induced arterial thrombosis rat model	Intravenous 1, 2, or 4 mg/kg single dose	↓ERK/NF-*κ*B pathway	No PK data	Qiu et al. (2020)
Vascular injury	TLFA	N/A	STZ-induced diabetic retinopathy rats	Oral 1.38, 0.69, or 0.35 g/kg/day × 9 weeks	↓VEGF and PKCβ2	Unstandardized extract; no PK data	Zhang et al. (2020)
Vascular injury	CADE	N/A	C57BL/6 mice fed an atherogenic diet with 10% fructose for 8 weeks	Oral 200 mg/kg/day × 8 weeks	↓VCAM-1	Unstandardized polyherbal; no PK data; single dose only	Song et al. (2018)
Vascular injury	ALP	HaCaT cells	Diabetic skin defect rat model	Topical 2% ALP-based hydrogel once daily	↓TNF-α, IL-6, and M1 macrophage polarization	Unstandardized botanical polysaccharide; no PK data	Xiang et al. (2025)
Vascular sclerosis	AREs	N/A	HFD-induced atherosclerosis Japanese quail model	Oral (four different AREs at 0.75–300 mg/kg/day) × 10 weeks	↓MDA; ↑SOD, CAT, GSH, NADPH, GSH-Px, and NO	Unstandardized multi-extracts; no PK data	Wang et al. (2016)
Endothelial injury	Arctigenin	PDGF-induced PASMCs	MCT-induced PAH rat model	Intraperitoneal 50 mg/kg/day × 28 days	↓NLRP3, caspase-1, and IL-1β	Single dose only; immune cells not assessed; no PK/PD data	Jiang et al. (2018)
Vascular injury	Arctigenin	N/A	SHRs, essential hypertension rat model	Oral 50 mg/kg/day × 8 weeks	↑eNOS/Akt ↓TXB2, SBP, and p47phox	Single dose only; sparse PK data; no causal proof	Liu et al., (2015b)
Vascular injury	Arctiin	N/A	STZ-induced rat model	IP 40 or 60 mg/kg/day × 8 weeks	↑Nephrin and podocin ↓Heparanase	No dose-response modeling; sparse PK data; no causal proof	Ma et al. (2013)
Vascular sclerosis	ALLE	ox-LDL-induced RAW264.7 macrophages	HFD and vitamin D_3_-induced atherosclerosis rat	Oral 100 or 200 mg/kg/day × 12 weeks	↓PI3K/AKT and NF-κB ↓VCAM-1, ICAM-1, and MMP-9	Unstandardized leaf extract; no PK data	Guo et al. (2024)

AG, arctigenin-4′-O-glucuronide; ALE, *A. lappa* root extract; ALLE, ethanolic extract of *A. lappa* leaves; ALPs, *Arctium lappa* polysaccharides; ALR-S, saccharides from *A. lappa* root; AREs, *A. lappa* root extracts; ARP, *A. lappa* root powder; CADE, a formulation containing *A. lappa* root extracts; CAT, catalase; CBM, carbomer 940; CMC, carboxymethyl cellulose; DNCB, 2,4-dinitrochlorobenzene; GSH, glutathione; GSH-Px, glutathione peroxidase; HAECs, human aortic endothelial cells; HFD, high-fat diet; HRCECs, human retinal capillary endothelial cells; HUVECs, human umbilical vein endothelial cells; hDPCs, human hair dermal papilla cells; IP, intraperitoneal injection; LPS, lipopolysaccharide; MCT, monocrotaline; MDA, malondialdehyde; NADPH, nicotinamide adenine dinucleotide phosphate; NO, nitric oxide; ox-LDL, oxidized low-density lipoprotein; PASMCs, pulmonary vascular smooth muscle cells; PBMCs, peripheral blood mononuclear cells; PDE4, phosphodiesterase-4; PK, pharmacokinetics; PO, *per os* (oral administration); SHRs, spontaneously hypertensive rats; SOD, superoxide dismutase; STZ, streptozotocin; TLFA, total lignans from Fructus arctii.

### 5.1 Effects of *A. lappa* extracts on AD

AD is a chronic relapsing inflammatory skin condition characterized by intense itching, skin barrier dysfunction, and immune dysregulation, particularly involving Th2 cytokines such as IL-4, IL-5, and IL-13. Conventional treatments, including topical corticosteroids and immunosuppressants, often cause long-term side effects (Alvarenga et al., 2024).

*A. lappa* extracts inhibit the NF-κB and MAPK signaling pathways, leading to reduced production of inflammatory cytokines and an alleviation of AD-associated skin inflammation (Sohn et al., 2011). In particular, butanol extracts of *A. lappa* (10–100 μg/mL) dose-dependently suppressed mast cell degranulation, whereas at 100 μg/mL, they inhibited IL-4 and IL-5 expression and secretion, along with NF-κB activation and MAPK phosphorylation in preclinical models (Sohn et al., 2011), and edible “Japanese leaf burdock” extracts (250–500 μg/mL) inhibited antigen-stimulated basophil degranulation *in vitro* (Maeta et al., 2021). Recent studies have further shown that oral administration of arctiin (25 mg/kg/day) inhibits inflammation and pyroptosis through the suppression of the TLR4/MyD88/NF-κB and NLRP3/Caspase-1/GSDMD signaling pathways in an AD murine model, positioning arctiin as a potential novel therapeutic agent for AD (Li J. et al., 2024). In addition, clinical case reports support the efficacy of Fructus Arctii-based herbal formulations in attenuating eczema severity and maintaining remission (Maraschio et al., 2023). Moreover, topical application of herbal formulations containing *A. lappa*, such as Derma-Hc, suppresses the expression and activation of IL-22, Janus kinase 1 (JAK1), and signal transducer and activator of transcription 3 (STAT3), resulting in marked reductions in pruritus, erythema, and epidermal hyperplasia in experimental models of AD (Nam et al., 2021). Other studies highlight the potential of arctigenin-containing natural mixtures in modulating the hypothalamic‒pituitary‒adrenal axis and decreasing Th2-driven inflammation (Nguyen et al., 2020). In addition, matairesinol, another lignan from *A. lappa*, exerted anti-allergic effects in a murine model of dermatitis (Sung et al., 2016), while distinct metabolomic profiles of *A. lappa* grown in European ecotypes have been identified, suggesting regional variability in bioactivity (Ambroselli et al., 2024).

Collectively, current evidence supports the use of *A. lappa* and its major lignans, especially arctiin and arctigenin, as complementary therapies for managing inflammation, allergic responses, and skin barrier dysfunction in patients with AD (Sohn et al., 2011). Although preclinical models consistently show that arctiin and arctigenin attenuate inflammatory pathways in AD, most studies rely on cell-based assays or small-scale animal models. The extracts used in these studies vary in composition and lack standardized quality control, which may limit reproducibility. Furthermore, clinical evidence is currently confined to case reports or small pilot trials, making it difficult to draw firm conclusions regarding efficacy and safety in humans. The lack of randomized controlled clinical trials and limited understanding of pharmacokinetics and skin permeability raise concerns about translational validity.

### 5.2 Effects of *A. lappa* extracts on psoriasis

Psoriasis is a chronic IMSD characterized by keratinocyte hyperproliferation and persistent inflammation driven by T cells and cytokines such as IL-17 and tumor necrosis factor (TNF)-α (Ma et al., 2024; Li L. et al., 2024). It has also been increasingly recognized that phytopharmaceuticals and bioactive metabolites derived from natural sources provide safe and promising alternative strategies for psoriasis management (Feng et al., 2025).

Arctigenin has demonstrated potent anti-psoriatic effects by inhibiting the NF-κB and MAPK pathways, reducing inflammatory cytokine production (Yinghua Ma, 2017) and regulating phosphodiesterase-4 (PDE4) activity. By inhibiting PDE4, arctigenin (10, 30, and 100 μM) increases intracellular cyclic adenosine monophosphate (cAMP) levels and activates the protein kinase (PKA)/cAMP response element-binding protein (CREB) signaling pathway, leading to the suppression of TNF-α and IL-6 production in keratinocytes (Li H. et al., 2021). Moreover, the topical application of arctigenin (5% cream) significantly reduces keratinocyte proliferation and immune cell infiltration in murine models of psoriasis, suggesting its potential as a novel topical agent for psoriasis treatment (Li H. et al., 2021). Additionally, arctigenin (10, 50, and 100 μM) modulates the keratinocyte cell cycle and apoptosis, addressing the excessive proliferation characteristic of psoriatic plaques (Du et al., 2016), and evidence from arthritis models further supports its potential application in treating psoriatic arthritis (Liu H. et al., 2015). Moreover, naturally occurring lignans, including those derived from *A. lappa*, efficiently induce apoptosis in colorectal tumor cells, which may also be relevant to hyperproliferative keratinocytes in psoriasis (Hausott et al., 2003).

These preclinical findings suggest that *A. lappa* and its lignans, including arctiin and arctigenin, hold promise as adjunct therapies to conventional biologics and immunosuppressants for the management of psoriasis. Although arctigenin demonstrates PDE4 inhibition and anti-inflammatory activity in murine psoriasis models, these findings have yet to be replicated in well-controlled human studies. The doses used in preclinical experiments are often much higher than what would be achievable in humans, raising questions about clinical translatability. Future studies should evaluate pharmacokinetics and optimize delivery systems to bridge this gap.

### 5.3 Effects of *A. lappa* extracts on SLE

SLE is a systemic autoimmune disease characterized by the aberrant activation of B cells, excessive production of autoantibodies, immune complex deposition, and chronic inflammation affecting multiple organs, including the skin, kidneys, joints, and central nervous system (Xiong et al., 2024). Dysregulated IFN-I signaling is recognized as a central driver of SLE pathogenesis. Recent studies have shown that arctigenin (15 mg/kg, intraperitoneally) attenuates SLE progression in murine models by inhibiting abnormal germinal center B-cell reactions and downregulating IFN-I signaling (Zhang X. et al., 2022).

Mechanistically, arctigenin activates protein phosphatase 2A (Guilbeau and Majumder, 2023), which leads to decreased phosphorylation of JAK1 and STAT1, resulting in the downregulation of IFN-α-stimulated gene expression. This action dampens autoantibody production and reduces systemic inflammation, highlighting the potential of arctigenin to modulate key pathogenic pathways involved in SLE. Moreover, oxidative stress contributes to SLE-related tissue damage (Yan et al., 2023). The antioxidant properties of arctigenin, including the suppression of ROS production and the promotion of cellular antioxidant defenses, may further protect against oxidative injury to endothelial cells and renal tissues in individuals with lupus nephritis, a severe complication of SLE (Lv et al., 2021; Miglioranza Scavuzzi and Holoshitz, 2022).

These findings suggest that arctigenin, which shows promise in preclinical lupus models by modulating IFN-I signaling and reducing autoantibody production, could serve as a promising adjunct therapeutic agent for SLE, addressing both immune dysregulation and organ protection. However, these findings are limited to murine studies. No randomized or controlled human trials have been conducted, and the immunological complexity of SLE raises concerns regarding translatability. In addition, most mechanistic studies focus on single signaling pathways, whereas SLE involves highly heterogeneous immune responses. There is a lack of standardized extract formulations and dose-finding studies, making evaluating their therapeutic potential difficult. Future work should prioritize multicenter animal studies with clinically relevant models, along with early-phase clinical trials, to assess safety, pharmacokinetics, and potential interactions with existing SLE therapies.

### 5.4 Effects of *A. lappa* extracts on alopecia

Alopecia encompasses a spectrum of hair loss disorders, including androgenetic alopecia and alopecia areata, which are often driven by oxidative stress, chronic inflammation, and immune-mediated follicular damage (Ma et al., 2020). ROS, especially hydrogen peroxide (H_2_O_2_), induces premature senescence and apoptosis in dermal papilla cells, which are crucial regulators of hair follicle growth (Wang et al., 2025).

Studies have demonstrated that arctiin protects human hair dermal papilla cells (hDPCs) from oxidative stress-induced cytotoxicity by inhibiting ROS production and preventing H_2_O_2_-triggered apoptosis (Bae et al., 2014). Moreover, in a well-established oxidative stress model, hDPCs were pretreated with purified arctiin (0–30 μM for 8 h) and then challenged with H_2_O_2_ (750 μM, 24 h). Arctiin at 10–30 μM preserved cell viability (minimum effective concentration ≈10 μM), reduced ROS accumulation, prevented G_2_/M arrest and sub-G1 enrichment, and decreased senescence-associated β-galactosidase activity, whereas pretreatment with 60 μM arctiin preserved cell cycle progression and reduced the expression of senescence markers, suggesting a role in maintaining hair follicle viability (Chowdhury et al., 2024). Mechanistically, arctiin modulates key signaling pathways involved in hair growth, including the MAPK and Wnt/β-catenin pathways (Bae et al., 2014). The activation of Wnt signaling promotes hair follicle stem cell activation and hair cycle progression, whereas the inhibition of stress-activated MAPKs (e.g., p38) prevents premature follicular senescence (Qiu et al., 2017). Moreover, the influence of arctiin on the expression profiles of miRNAs (e.g., miR-31 and the miR-200 family) suggests an additional epigenetic mechanism through which it may regulate DPC survival and function (Bae et al., 2014). Given the limited efficacy and potential side effects of current alopecia treatments, such as minoxidil and corticosteroids, arctiin may be a promising natural alternative that targets the oxidative and inflammatory pathways that are central to hair follicle health.

Although arctiin protects dermal papilla cells from oxidative stress-induced senescence and apoptosis, current evidence remains limited to *in vitro* systems or short-term oxidative stress models. There is no *in vivo* animal model evidence directly linking *A. lappa* extracts to hair regrowth, and no human clinical data exist. Furthermore, the concentrations of arctiin used in experimental studies may not be achievable *in vivo* without advanced delivery strategies. Another limitation is that the potential endocrine and immune-mediated pathways in alopecia are largely unexplored in relation to *A. lappa*. Future research should focus on validating these findings in established alopecia models, examining long-term follicular biology, and conducting controlled clinical studies to determine efficacy and safety.

### 5.5 Effects of *A. lappa* extracts on SSc

SSc is a complex systemic autoimmune disease characterized by progressive fibrosis of the skin and internal organs, vascular abnormalities, and immune dysregulation (Sierra-Sepúlveda et al., 2019). The triad of autoimmunity, microangiopathy, and fibroblast activation underlies its pathogenesis.

Disruptions in adiponectin signaling are implicated in the development of fibrosis and vascular dysfunction in individuals with SSc (Ko et al., 2023). Through its receptors AdipoR1 and AdipoR2, adiponectin exerts anti-inflammatory, anti-fibrotic, and vasculoprotective effects (Aljafary and Al-Suhaimi, 2022). Notably, reduced adiponectin levels are correlated with disease severity and the extent of fibrosis. Additionally, arctiin and arctigenin have been identified as natural AdipoR1 agonists capable of enhancing adiponectin signaling (Yamashita et al., 2018; Sun et al., 2013). Arctiin and its aglycone, arctigenin, were identified as agonists of AdipoR1 (Sun et al., 2013). Since adiponectin exerts anti-fibrotic effects by activating AdipoR1/AMPK signaling and downregulating canonical TGF-β/Smad pathways in fibroblasts (Fang et al., 2012), these metabolites may also attenuate fibroblast-to-myofibroblast differentiation and extracellular matrix deposition. Furthermore, by stabilizing dermal white adipose tissue, preventing adipocyte loss, and reducing inflammatory cytokine release (e.g., IL-6 and IL-1β), arctiin may help preserve skin architecture and vascular function in individuals with SSc (Wu et al., 2025). Recent studies further indicate that adiponectin receptor agonists, including natural metabolites such as arctiin and arctigenin, not only reduce fibroblast activation but also may enhance the efficacy of established anti-fibrotic therapies (Chen T. et al., 2024). This highlights the potential of multitargeted approaches in SSc, where simultaneous modulation of adiponectin signaling and other profibrotic pathways could yield synergistic therapeutic benefits. Thus, arctiin and arctigenin represent promising candidates for future clinical trials aimed at mitigating fibrosis and immune dysregulation in SSc.

Although preclinical studies suggest that arctiin and arctigenin act as natural AdipoR1 agonists with anti-fibrotic effects, the evidence is largely derived from cell-based assays and murine fibrosis models. The pathology of human systemic sclerosis is complex and involves both immune dysregulation and microvascular abnormalities, which are not fully understood in animal models. Moreover, current studies often use high doses of isolated metabolites that may not reflect achievable plasma levels in patients. Clinical data are completely absent, and standardized extract formulations have not been tested in SSc patients. To enhance translational potential, rigorous pharmacokinetic studies, safety assessments, and early-phase clinical trials are needed to determine whether the anti-fibrotic effects observed in preclinical research can be replicated in patients.

### 5.6 Effects of *A. lappa* extracts on vasculitis

Vasculitis encompasses a heterogeneous group of disorders characterized by inflammation and necrosis of the blood vessel wall, affecting vessels of various sizes and leading to tissue ischemia and organ dysfunction (Morita et al., 2020). Inflammatory cytokines (IL-6, IL-8, IL-1β, and TNF-α) and angiogenic factors, such as vascular endothelial growth factor (VEGF), play central roles in vasculitic processes by promoting endothelial activation, leukocyte adhesion, and vascular damage (Zhou et al., 2020; Zhang et al., 2020). Activation of the NF-κB pathway further amplifies the inflammatory response (Zeng et al., 2024).

In a TNF-α-induced early atherosclerosis model characterized by vascular inflammation, *A. lappa* root extract (ALE, 100 mg/kg/day) reduced monocyte infiltration and suppressed VCAM-1 expression in the aortic root *in vivo* (Lee et al., 2020). Consistently, in endothelial cells, ALE (25–100 μg/mL) inhibited NF-κB signaling and decreased VCAM-1 and pro-inflammatory cytokine expression, indicating its potential relevance for attenuating endothelial activation and leukocyte recruitment in cutaneous vasculitis (Lee et al., 2020). Moreover, by inhibiting the NLRP3 inflammasome and reducing ROS production, these metabolites decrease endothelial oxidative stress, preserving vascular integrity (Bai et al., 2020). This phenomenon is particularly relevant in anti-neutrophil cytoplasmic antibody (ANCA)-associated vasculitis, Takayasu arteritis, and giant cell arteritis, in which oxidative stress and endothelial dysfunction are prominent pathogenic features (Ecclestone and Watts, 2023). Collectively, the multifaceted anti-inflammatory and endothelial-protective actions of arctiin and arctigenin position them as potential adjunct therapies for vasculitis.

However, evidence for the efficacy of arctiin and arctigenin in vasculitis is currently indirect and derived from endothelial cell assays and animal models of vascular inflammation or atherosclerosis. Although these studies suggest the suppression of NF-κB activation, a reduction in adhesion molecule expression, and protection against oxidative stress, none have been performed in established models of autoimmune or ANCA-associated vasculitis. Thus, extrapolation to human vasculitis should be made with caution. Furthermore, variability in extract composition and a lack of standardized dosing regimens limit comparability across studies. The absence of clinical evidence is a major gap. Future research should establish disease-relevant animal models of vasculitis, evaluate long-term vascular outcomes, and ultimately conduct pilot clinical studies before therapeutic applications are considered.

## 6 Future directions and clinical perspectives

Arctiin and arctigenin have shown significant therapeutic potential across various immune-mediated and inflammatory contexts, suggesting promising avenues for the management of IMSDs. Research highlights the ability of arctiin to suppress key inflammatory pathways, such as the TLR4/MyD88/NF-κB and NLRP3/Caspase-1/GSDMD signaling pathways, which are critically involved in immune dysregulation and pyroptosis and play a role in the progression of IMSDs such as AD. In AD models, arctiin effectively reduces skin lesion severity and inflammation, suggesting its potential as a safer alternative to conventional corticosteroids and immunosuppressants (Li J. et al., 2024).

Similarly, arctigenin exerts robust anti-inflammatory and immunomodulatory effects by modulating the PI3K/AKT/mTOR (Chen X. F. et al., 2024) and Wnt/β-catenin pathways (Yoo et al., 2010). Arctigenin also targets key regulators, such as mitogen-activated protein kinase kinase 1 (MEK1) and peroxisome proliferator-activated receptor gamma (PPARγ), which are functionally associated with Wnt signaling. These pathways and molecules play critical roles in regulating inflammation, cellular metabolism, and immune homeostasis. The ability of arctigenin to inhibit epithelial‒mesenchymal transition and suppress cytokine release makes it a promising agent for mitigating tissue inflammation and fibrosis, which are common complications of chronic autoimmune diseases (Chen X. F. et al., 2024; Li H. et al., 2024). In addition to immune modulation, both arctiin and arctigenin exhibit potent antioxidant properties, reducing the oxidative stress that perpetuates chronic inflammation in IMSDs (Jia et al., 2024). Addressing oxidative stress is crucial as it plays a central role in sustaining the inflammatory cycle and tissue damage under these conditions.

Despite the encouraging preclinical data, several key challenges must be addressed before *A. lappa*-derived metabolites can be translated into clinical applications. First, most evidence supporting the efficacy of arctiin and arctigenin comes from *in vitro* experiments or animal models, which limits direct clinical relevance. Well-designed clinical trials are urgently needed to validate their therapeutic benefits, optimize dosing regimens, and confirm long-term safety profiles. Phase I trials should establish the safety, tolerability, and pharmacokinetics of standardized *A. lappa* extracts and purified lignans in healthy volunteers. Subsequent phase II proof-of-concept studies in patients with IMSDs should focus on validated endpoints such as the eczema area and severity index (EASI), the psoriasis area and severity index (PASI), pruritus intensity, quality-of-life indices, and cytokine or transcriptomic biomarkers. Ultimately, phase III multicenter randomized controlled trials will be essential to compare *A. lappa*-derived interventions with current standard-of-care therapies, with endpoints including durable disease control, relapse prevention, and steroid-sparing effects. These steps will be critical for establishing clinical evidence for the therapeutic potential of *A. lappa* in IMSDs. Second, the pharmacokinetics and bioavailability of arctiin and arctigenin require further elucidation. These metabolites may undergo extensive metabolism following oral administration, which potentially limit their systemic bioactivity. Advanced drug delivery strategies, including nanoparticle-based formulations or transdermal systems, could increase their bioavailability and facilitate targeted delivery to inflamed tissues. Third, although the suppression of specific inflammatory pathways, such as the NF-κB and JAK/STAT pathways, has been documented, the broader molecular interactions and network effects of arctiin and arctigenin remain incompletely characterized. Integrative omics approaches involving transcriptomics, proteomics, and metabolomics could reveal the full spectrum of biological activities and identify biomarkers predictive of a therapeutic response.

In terms of safety, preliminary studies suggest that *A. lappa* root extracts are well tolerated. Oral administration of its aqueous extract at doses up to 250 mg/kg/day for 8 weeks in mice did not result in observable organ toxicity or hematological abnormalities (Bok et al., 2017), supporting its development as a functional food ingredient or medicinal resource. Nevertheless, comprehensive toxicological assessments, including evaluations of genotoxicity, reproductive toxicity, and immunotoxicity, are essential to ensure clinical safety.

Future research should prioritize rigorous preclinical and clinical studies to establish the therapeutic value of *A. lappa*-derived metabolites. Although arctiin and arctigenin are the most extensively studied lignans (Ziętal et al., 2024), the pharmacological potential of *A. lappa* likely extends beyond these metabolites to include flavonoids, polysaccharides, and other secondary metabolites. Broader investigations into the full spectrum of bioactive metabolites, alongside continued work on well-characterized lignans, will be critical for achieving a more comprehensive understanding of their pharmacology. In parallel, advances in pharmacogenomics and systems biology may, in the long term, support more refined patient stratification and personalized applications of *A. lappa*-based botanical drugs.

In conclusion, arctiin and arctigenin represent promising natural modulators of immune and inflammatory pathways. Strategic, multidisciplinary research efforts integrating pharmacology, clinical medicine, formulation science, and systems biology will be essential to fully harness their therapeutic potential and advance them as safe, effective options for the management of IMSDs.
