# Supplementary material for: Immunopharmacological potential of Arctium lappa L. in immune-mediated skin diseases: A critical review of experimental and clinical evidence
Source: Front Pharmacol. 2025 Oct 3;16:1660352. doi: 10.3389/fphar.2025.1660352 (PMC12531496; doi:10.3389/fphar.2025.1660352)
Supplement: Supplementary file 1 [file Table1.pdf]

Supplementary Table S1. Synonyms of *A. lappa* according to POWO

Synonym	Original Reference	Note
Arctium lappa subsp. *majus* Arènes	Bull. Jard. Bot. État Bruxelles 20: 75 (1950)	not validly publ.
Arctium majus Bernh.	Syst. Verz. Erf.: 154 (1800)	nom. superfl.
Arctium ruderale Salisb.	Prodr. Stirp. Chap. Allerton: 184 (1796)	nom. superfl.
Lappa arctium Hill	Veg. Syst. 4: 51 (1762)	—
Lappa bardana Moench	Methodus: 552 (1794)	nom. superfl.
Lappa bardana var. *major* Čelak.	Prodr. Fl. Böhmen: 249 (1871)	not validly publ.
Lappa communis Coss.	Fl. Descr. Anal. Paris: 389 (1845)	nom. superfl.
Lappa communis var. *major* Coss.	Fl. Descr. Anal. Paris: 389 (1845)	not validly publ.
Lappa lappa (L.) H. Karst.	Deut. Fl.: 1121 (1883)	not validly publ.
Lappa major Gaertn.	Fruct. Sem. Pl. 2: 379 (1791)	nom. superfl.
Lappa officinalis All.	Fl. Pedem. 1: 145 (1785)	nom. superfl.

Source: Plants of the World Online (POWO), Kew Science. Accessed September 2025.
